# Serum Hyaluronic Acid as a Noninvasive Marker of Hepatic Fibrosis in Chronic Hepatitis B

**DOI:** 10.4103/1319-3767.43274

**Published:** 2008-10

**Authors:** Bita Geramizadeh, Katayoun Janfeshan, Mehdi Saberfiroozi

**Affiliations:** Department of Pathology, Transplant Research Center, Gastroenterology Research Center, Shiraz University of Medical Sciences, Shiraz, Iran

**Keywords:** Hepatic fibrosis, hepatitis B, hyaluronic acid

## Abstract

**Background/Aims:**

Chronic hepatitis B is a serious global health problem. Liver biopsy is currently recommended as the gold standard for the evaluation of the degree of fibrosis in patients with chronic hepatitis B. This procedure, however, is invasive and has potential complications. In this study, we attempted to validate the level of hyaluronic acid as a simple laboratory test to discriminate between patients with and without significant fibrosis in chronic hepatitis B.

**Methods:**

This study included 93 patients with chronic hepatitis B who had undergone percutaneous liver biopsy from 2003 to 2006. At the time of biopsy, a sample of serum was taken for the hyaluronic acid (HA) assay. Histological assessment consisted of the semiquantitative analysis of the degree of fibrosis according to the criteria proposed by the Ishak system. These findings were then compared by using statistical analysis.

**Results:**

HA levels and stage groups of fibrosis were well correlated (Spearman r = 0.945, *P* < 0.005). There was a significant increase in HA levels when considering S0 to S6. The mean values of HA concentrations were 59.7 ± 10.5 ng/mL for stages 0–2, 149.4 ± 15.9 ng/mL for stages 3–4 , and 284.5 ± 14.5 ng/mL for the last group (stages 5-6). There were significant differences between the three groups. Serum HA levels of cases with extensive fibrosis were significantly higher than in those with mild and moderate fibrosis (*P* = 0.0001, *P* = 0.0005, and *P* = 0.0001, respectively).

**Conclusion:**

Serum HA level is a precise predictor of extensive liver fibrosis in chronic hepatitis B. HA is well correlated with the stage of fibrosis and can reflect the severity of fibrosis. Thus, it can be used as a noninvasive test to monitor these patients.

Chronic hepatitis B virus affects 350 million individuals worldwide,[1,2] and hence, it is crucial to monitor the course of this disease more closely. Hepatic fibrosis has been a common response to chronic liver injury and might result in potentially lethal sequelae. In chronic liver diseases, both the determination of the stage of the fibrotic process and evaluation of antifibrotic treatment require accurate variables. Hence, it would be of great value to explore a credible, specific, and noninvasive diagnostic parameter of liver fibrosis for the prevention and treatment of chronic liver disease. Liver biopsy is currently recommended as the gold standard for the staging of fibrosis in patients with chronic hepatitis B. The risk of developing cirrhosis depends on the stage (degree of fibrosis) and the grade (degree of inflammation and necrosis) observed in the initial liver biopsy. This procedure, however, is invasive and has potential complications. Noninvasive approaches developed to assess histological samples include clinical symptoms, routine laboratory tests, and radiographic imaging, but all of them have poor correlation with histology. Because of the limitations of conventional approaches, several noninvasive tests have been developed for this purpose.[3,4] Several clinical studies have attempted to identify serum markers that correlate with the degree of fibrosis and its feasibility, in conjunction with or in place of a liver biopsy, such as the measurement of substances that regulate fibrosis or participate in the generation of the liver's extracellular matrix.[2,5,6] Among these, the majority of reports and studies are about hyaluronic acid (HA), type IV collagen, N-terminal propeptide of type III procollagen, metalloproteinases, inhibitors of metalloproteinases, and transforming growth factor beta (TGF-β).[7–9] HA is a high-molecular-weight glycosaminoglycan, which is an essential component of extracellular matrix in virtually every tissue in the body.[6] Currently, it has been introduced as one of the best available markers of hepatic fibrogenesis in chronic viral hepatitis.[8]

In this study, we aimed to find out the utility of serum hyaluronic acid levels as a predictive marker of liver fibrosis in the patients with chronic hepatitis B.

## PATIENTS AND METHODS

### Study population

Our study population consisted of 93 patients (63 male and 30 female) with Hepatitis B surface antigen (HBS) positive chronic hepatitis B, aged between 23 and 55 years. Patients with autoimmune hepatitis or HCV coinfection were excluded from the study by assaying for HCV and antinuclear antibodies. None of the patients was treated with antiviral agents during the 12-month period before inclusion into the study. None of them had any evidence for decompensated cirrhosis or any other causes of chronic hepatitis. A 5-mL sample of peripheral blood was taken from each patient one hour before performing liver biopsy. The serum was isolated and kept at -70°C for the measurement of serum hyaluronic acid levels.

### Measurement of hyaluronic acid

Hyaluronic acid (HA) test kit was provided by Corgenix Inc. (Colorado, USA, under license of Chugai diagnostic science Co.), and the serum HA was measured by ELISA according to the manufacturer's instructions. A 100-*μ*L aliquot of serum or reference solution (dilution 1/10) was added to each hyaluronic-binding protein (HABP)-coated microwell and incubated for 30 minutes. Subsequently, 100 *μ*L of stop solution (0.36 N sulfuric acid) was added. The HA concentration was assayed by using an enzyme-linked binding protein assay that used HABP as the capture molecule. All HA assay results were obtained within a week after liver biopsy.

None of the patients had any concurrent liver disease such as alcoholic hepatitis or HCV. Also, none of them had received any antiviral treatment before liver biopsy and sample collection.

Hematoxylin and eosin (H&E) and Masson-Trichrom stained specimens were observed by two pathologists (a hepatopathologist and a general pathologist) for all the biopsies. Both of them were completely unaware of the clinical and paraclinical findings. Biopsies that were < 2 cm in size were excluded from the study.

Liver biopsy grading and staging were done according to the modified HAI (Ishak's) method. As we did not have any information about the patients' body mass index to exclude obesity, we excluded all the liver biopsies that showed any evidence of steatosis.

The association between HA levels and liver biopsy staging was measured with the Mann-Whitney test and with the Spearman rank correlation coefficient. ***P*** < 0.05 was considered to be statistically significant.

## RESULTS

Of the 93 patients selected, 54 (58.1%) presented with no fibrosis (S0), only some had portal fibrous expansion (S1) or at the most portal fibrous expansion (S2) detected during histological examination, whom we categorized as group 1 (mild fibrosis). Twenty-four (25.8 %) patients showed fibrous expansion of portal areas with P-P bridging (S3) or marked fibrosis of portal areas with P-P and P-C bridging (S4), and they were classified as group 2 (moderate fibrosis). Fifteen (16.1%) patients showed structural alterations that were characterized by the presence of marked bridging and nodule formation (S5) or cirrhosis (S6) and were classified as group 3 (severe fibrosis and cirrhosis) [Table 1].

**Table 1:** Frequency of each group of the patients

Different stages	Frequency	Percent
S0, S1, S2	54	58
S3, S4	24	25.8
S5, S6	15	16.1
Total	93	100

HA levels and stage groups of fibrosis were well correlated (Spearman r = 0.945, ***P*** < 0.005) and there was a significant increase in HA levels when considering S0 to S6. The mean values of HA concentrations were 59.7 ± 10.5 ng/mL for stages 0–2, 149.4 ± 15.9 ng/mL for stages 3–4, and 284.5 ± 14.5 ng/mL for stages 5–6 [Table 2].

**Table 2:** 95% Confidence interval of mean values of hyaluronic acid for predicting the three stages

Stages	S0–S3	S3, S4	S5, S6
95% confidence interval			
Lower bound	49.30	133.56	270.00
Upper bound	70.27	165.37	299.15
Mean	59.78	149.47	284.58

**Figure 1:**
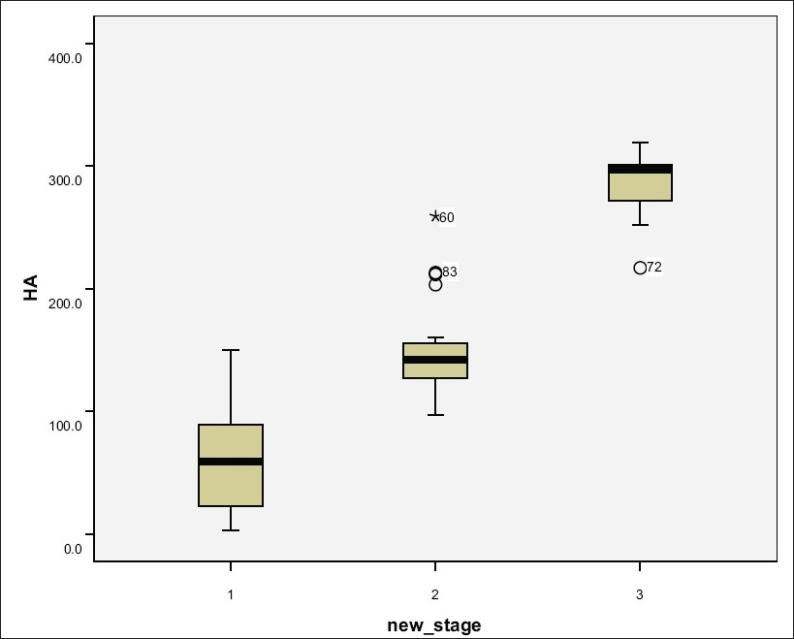
HA concentrations of patients' groups

**Figure 2:**
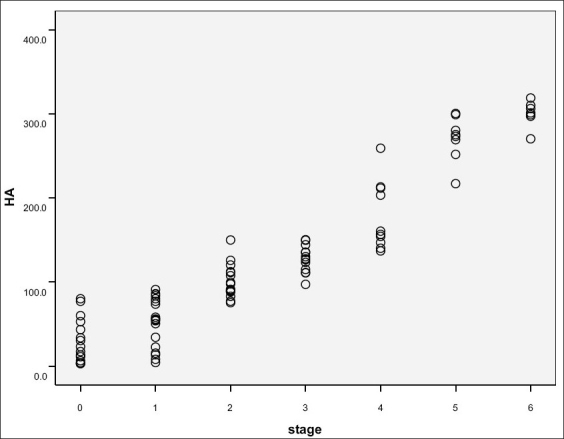
HA distribution in different stages

Stagewise distribution of HA concentrations is shown in Figures 1 and 2. Patients with chronic hepatitis B had higher serum hyaluronate levels than normal subjects (75 ng/mL).

We observed great differences in the serum HA levels between the three groups in our study. The number of cases with extensive fibrosis was significantly greater than those with mild or moderate fibrosis (***P*** = 0.0001, ***P*** = 0.0005, and ***P*** = 0.0001 respectively). A cutoff value of 113 ng/mL was chosen for identifying the absence or presence of mild fibrosis (S0–S2). The presence of moderate to severe fibrosis could be excluded with a high certainty by applying the cutoff point, as only three (5%) of the 54 patients with HA levels < 113 ng/mL had moderate to severe fibrosis, with a negative predictive value (NPV) of 89% and positive predictive value (PPV) of 94%. A cutoff value of 181.9 ng/mL was chosen for identifying the absence or presence of severe fibrosis and cirrhosis (S5–S6). Applying the cutoff point, none (NPV of 100%) of the 74 patients with HA < 181.9 ng/mL had cirrhosis; a PPV of 78% was obtained for predicting the presence of severe fibrosis and cirrhosis [Table 3].

**Table 3:** Sensitivity, specificity, negative and positive predictive value of the patients with HA < 113 ng/mL and HA > 181 ng/mL to predict no significant fibrosis and severe fibrosis respectively

	Sensitivity	Specificity	NPV	PPV
HA < 113 ng/mL	92%	95%	89%	94%
HA > 181 ng/mL	100%	95%	100%	78%

NPV- Negative predictive value, PPV- Positive predictive value, HA - Hyaluronic acid

## DISCUSSION

Chronic injury leading to fibrosis in the liver occurs in response to a variety of pathologies, including viral hepatitis (especially hepatitis B and C), alcohol abuse, schistosomiasis, drug toxicity, metabolic diseases due to overload of iron or copper, autoimmune attack of hepatocytes or bile duct epithelium, or congenital abnormalities. Liver fibrosis is reversible whereas cirrhosis, the end-stage consequence of fibrosis, is generally irreversible.[1]

Noninvasive evaluation of liver fibrosis is thus of great clinical interest. Many parameters for noninvasive diagnosis of liver fibrosis have been studied extensively in the past, but none has yet replaced liver biopsy as the gold standard.[2]

Ideal markers of fibrosis should have a high degree of sensitivity and specificity, be easily measured and reproducible, readily available, inexpensive, and useful in accurately following the disease progression. The diagnostic panel in its current form simply provided a binary distinction between no or mild and advanced fibrosis that could provide additional and potentially important prognostic information. Reducing the requirement of liver biopsy prior to initiating treatment would be an important factor in improving the cost-effectiveness and risk-benefit ratio in the management of chronic hepatitis B and C patients.[3]

In this study, we attempted to validate HA as a simple laboratory test to discriminate between patients with and without significant fibrosis in a consecutive series of chronic hepatitis B patients. The high level of confidence for excluding significant fibrosis was the same as those of many other studies.[1,2]

In the present study, HA levels were accurate in predicting mild, moderate, and severe fibrosis, as well as cirrhosis. Therefore, it is likely that the association of HA levels with the degree of hepatic fibrosis represents an indirect one, expressing the functional correlation between fibrosis and both concomitant capillarization and hepatic hemodynamic changes. Mild fibrosis can be predicted by HA levels < 113 ng/mL for both its absence (NPV of 89%) and for its presence (PPV of 94%). Severe fibrosis and cirrhosis can be predicted by HA levels > 181.9 ng/mL for both its absence (NPV of 100%) and its presence (PPV of 78%).

Serum hyaluronate concentrations have been reported to have a high sensitivity and an acceptable false-negative rate for the diagnosis of compensated cirrhosis. Therefore, serum HA concentrations can be considered as a diagnostic variable for compensated cirrhosis.[5] Simultaneous determination of the levels of serum HA, collagen IV, and laminin in posthepatitic cirrhosis cases and chronic hepatitis cases has been reported to enhance diagnostic sensitivity but decrease the diagnostic specificity.[6] Among 13 variables associated with liver fibrosis selected by univariate analysis in chronic hepatitis B patients, age, γ-glutamyltranspeptidase (GGT), hyaluronic acid (HA), and platelet counts (PLT) were identified by multivariate logistic regression analysis as independent factors of fibrosis. A fibrosis index was constructed from the above four markers. By using an optimal cutoff score of 3.0, the sensitivity of the index is 90.2%, the specificity 76.1%, and the accuracy 82%. There was a positive linear relationship between the index scores and the stages of fibrosis (***P*** < 0.001).[7]

Most of the literature reports regarding chronic hepatitis C and hyaluronic acid concentration as its marker are also promising. In a study by Fontana ***et al.***,[10] simultaneous determination of HA, TIMP-1, and platelet counts were found to be well correlated with the degree of fibrosis. In another study from Egypt, lower levels of HA showed a good correlation with a low risk of fibrosis in young patients with chronic hepatitis C.[11]

Jun Tao and Hui-Qin Peng reported that HA is the most ideal index for diagnosing hepatic fibrosis. This was consistent with the results of their previous study on the relationship between serum fibrosis indices and liver histological changes. This suggests that the four serum fibrosis indices (HA, procollagen III, LN, and collagen IV) are influenced by inflammation which is scored by grade. In patients with chronic hepatitis, it is possible that the four serum fibrosis indices can be inconsistent with higher stages of hepatic fibrosis such as S3 or S4, and the grade is at low levels,. It was also suggested that these four serum fibrosis indices could be lowered by antiinflammatory treatment in patients with chronic hepatitis. They concluded that serum fibrosis indices can only reflect abnormal metabolism of the extracellular matrix. These indices are nonspecific biochemical markers, when they are used in the diagnosis of hepatic fibrosis, other diseases should be ruled out. They should not be called hepatic fibrosis markers, serum fibrosis indices should be the appropriate term.[12,13]

Montazeri ***et al.*** studied HA concentrations in serum of HBeAg-negative patients and showed that patients with chronic hepatitis B had higher serum HA levels than normal volunteers (73.4 ± 124.8 vs 20.4 ± 15.5 ng/mL respectively; ***P*** = 0.002). Serum HA levels of cases with extensive fibrosis were significantly higher than those with mild fibrosis (309.7 ± 143.5 vs 24.7 ± 31.9 ng/mL respectively; ***P*** < 0.001). Fifty-four out of 65 patients with chronic hepatitis B with mild inflammation (grades 0–8) had HA levels of 39.6 ± 74.2 ng/mL, while 11 of them with severe inflammation (grade = 9) had serum HA levels of 236.5 ± 188.7 ng/mL (***P*** = 0.006).[1]

Thus, in clinical practice, liver function, case history, and clinical manifestations should all be considered in the assessment of the diagnostic value of these fibrosis indices in the staging of hepatic fibrosis.

Unlike previous reports, we could not differentiate among individual Ishak fibrosis stages in this study.[12] Another limitation of using HA concentration is its increase secondary to active rheumatologic conditions.[12] Although we did not have such patients, this secondary increase should be considered in the evaluation of HA levels in patients with chronic hepatitis B.

While assessing the therapeutic efficacy of antifibrosis drugs, a comprehensive analysis is necessary instead of only depending on these four fibrosis indices.[11] To evaluate the antifibrotic effect of interferon α (INF-α ) and to study the relationship between INF-α and serum fibrotic markers, the posttreatment levels of HA, PC-III, and TGF-β-1 were all significantly lower than those before treatment (***P*** < 0.01)[13]

According to our study, which has included 93 patients with chronic hepatitis B, HA levels can be used for predicting the degree of fibrosis and also excluding significant fibrosis, which is very important especially in countries with high numbers of hepatitis B patients.
